# Linking policy and practice in monitoring socially responsible research and innovation (RRI): A conceptual framework to evaluate progress through the UNESCO-led Recommendation on Science and Scientific Researchers

**DOI:** 10.12688/openreseurope.14263.2

**Published:** 2023-07-24

**Authors:** Eric Allen Jensen

**Affiliations:** 1 Thriving Natural Capital Challenge Centre, SRUC, Edinburgh, Scotland, UK; 2 Institute for Methods Innovation, Dublin, Ireland

**Keywords:** RRI, socially responsible science, researchers, scientists, indicators, evaluation

## Abstract

This paper sets out a high-level conceptual framework for monitoring the development of socially responsible research and innovation (RRI) systems linked to the global policy instrument called ‘the Recommendation on Science and Scientific Researchers’ (RSSR). RRI is an umbrella concept, rooted in EU policy and research, that encompasses a range of topics that overlap directly with the RSSR. These topics include social inclusion in scientific research, improved scientific transparency and governance and expanded public engagement with science. The RSSR is an international accord ratified by 195 United Nations Educational Scientific and Cultural Organization (UNESCO) Member States in 2017, updating an earlier version of the instrument. This UNESCO-led initiative offers a globally inclusive and agreed structure for advancing RRI-related principles. A key feature of the RSSR initiative is its permanent structure of quadrennial monitoring to assess implementation of its principles. Here, a conceptual framework is presented to clarify the specific dimensions of RRI embedded in the 10 key priority areas for this quadrennial monitoring process. The paper explicates these dimensions and the underpinning policy language from the 2017 recommendation with the aim of supporting UNESCO Member States and research stakeholders globally to design appropriate evaluation methods. This conceptual framework is intended to support development of globally aligned measurement of RRI policy and practice that allows research and policy stakeholders from each world region to learn from each other. Fostering mutual learning on a global scale will help to enable evidence-based practice in the context of RRI to improve outcomes and mitigate the limitations of well-meaning but ineffective policies and practices.

## Disclaimer

The views expressed in this article are those of the author. Publication in on Open Research Europe does not imply endorsement by the European Commission.

## Introduction

The present text puts forward an overarching conceptual framework to support monitoring of an international accord adopted by 195 governments, representing a global commitment to socially responsible research and innovation (RRI). This accord sets out a wide range of principles for regulatory action on RRI, explicitly committing governments to take pro-RRI actions. The UN-organized accord is called the ‘Recommendation on science and scientific researchers (2017)’ (RSSR) ( UNESCO, 2017). This paper aims to bolster the RSSR’s role as a global vehicle for RRI by providing a conceptual framework for its associated quadrennial monitoring process.

RRI is an umbrella concept rooted in EU policy and research, that encompasses a range of topics that overlap directly with the RSSR ( Gerber *et al.*, 2020). These topics include social inclusion in scientific research, improved scientific transparency and governance and expanded public engagement with science. The paper explicates the dimensions of the RSSR that have been identified as priorities for measurement by the United Nations Educational Scientific and Cultural Organization (UNESCO), and the underpinning policy language from the 2017 recommendation. This elaboration of the existing guidance from UNESCO for the monitoring process is aimed at supporting UNESCO Member States and research stakeholders globally to design appropriate evaluation methods. This is because good evaluation methods require clear elaboration of the outcomes that are being targeted for measurement ( Jensen, 2014; Jensen & Laurie, 2016). Such evaluation can lay the foundations for long-term international mutual learning at the level of best practices in RRI advancement and evaluation, linked to the RSSR’s quadrennial cycle of stakeholder consultation, monitoring, and reflection at the national level. This monitoring process ( UNESCO, 2021) is a legal expectation for each UNESCO Member State to provide national reporting on progress towards the RSSR’s full implementation with appropriate substantiation, along the same lines as other UN conventions ( UNESCO, n.d.). Indeed, having comparable assessments conducted across countries and time can be highly useful for policymakers as they consider the common global standards embedded in the RSSR, along with analysis of local research and innovation policies and practices that can deliver on those standards.

## Context for conceptual framework

This conceptual framework is aimed at supporting each government and scientific community across the UNESCO Member States to take necessary actions for effective evaluation and reporting on RRI dimensions during their ongoing engagement with this quadrennial process. The tone and structure of this open letter is designed to be helpful to these research and policy stakeholders as they come to grips with the RSSR and its monitoring process. To aid the process of measuring progress in addressing the different dimensions included in the RSSR, this paper provides a conceptual framework with a detailed identification of the specific elements of the 10 key priority areas for monitoring the RSSR. The 10 priority areas that have been identified by UNESCO and confirmed by Member States in the UNESCO Executive Board in March 2020 ^1^ ( UNESCO, 2020) as the initial focus for RSSR implementation and monitoring are outlined in Table 1. The specifics of how this set of 10 key priority areas was initially fashioned have not been publicized by UNESCO beyond indicating that the goal was to achieve simplification to ease the burden of the monitoring process.

**Table 1.** 10 key priority areas identified by UNESCO and confirmed by Member States as the initial focus for RSSR implementation and monitoring. ( UNESCO, 2017).

**1. Responsibility of science towards the United Nations’ ideals of human dignity, progress, justice, peace, welfare** **of humankind, and respect for the environment.**
**2. Need for science to meaningfully interact with society and *vice versa*.**
**3. Role of science in national policy and decision- making, international cooperation, and development.**
**4. Promotion of science as a common good.**
**5. Inclusive and non-discriminatory work conditions and access to education and employment in science.**
**6. Any scientific conduct is subject to universal human rights standards.**
**7. Balancing the freedoms, rights, and responsibilities of researchers.**
**8. Scientific integrity and ethical codes of conduct for science and research and their technical applications.**
**9. Importance of human capital for a sound and responsible science system.**
**10. Role of Member States in creating an enabling environment for science and research.**

This open letter takes these confirmed key priority areas for the RSSR as a starting point and uses a close reading of the original RSSR to break each priority area down into its component dimensions to clarify where measurement is needed to feed into the long-term monitoring process for the RSSR (see supplemental materials for details). To further guide UNESCO Member States and research stakeholders in their consideration of relevant evaluation measures and indicators, guidance notes, and direct quotations from the RSSR are included in grey font in the full framework document (see underlying data: Jensen, 2021). These notes are focused on dimensions where relevant measurement options may be more ambiguous or needing elaboration to identify the relevant aspects to target for RSSR reporting. This presentation of the 10 key priority areas for RSSR implementation and monitoring uses verbatim language from UNESCO policy documents extensively to provide assurance of the alignment of the conceptual framework to the underpinning policy instrument. The conceptual framework presented is one of a series of publications intended to guide UNESCO Member States through the process of evaluating RRI progress in science systems ( Jensen, 2020). This evaluation process is required by the RSSR, but the specifics of how to assess and improve the socially responsibility of science systems in line with this policy instrument are not spelled out by UNESCO or the UNESCO Executive Board. This means that many countries’ representatives are left without detailed advice about how to interpret, implement, and evaluate the RSSR in their national systems. In part, this ambiguity is by design because it allows for context-appropriate adaptation of general RRI principles. Indeed, national governments are encouraged, but not required, to convene ‘working groups’ to provide diverse stakeholder voices, including representatives from scientific bodies, industry, citizen groups, etc., for this process of adaptation. However, greater elaboration and clarification of the components of the monitoring framework can streamline the initial steps of coming to grips with the policy and its dimensions, thus improving the experience for national governments and boosting the value of the monitoring exercise.

## Development of the conceptual framework

The conceptual framework presented in this paper was developed during the responsible research and innovation networked globally (RRING) (rring.eu) project to guide the project’s work in conducting three national case studies in South Africa, Lithuania, and Serbia focusing on the monitoring process for the RSSR. UNESCO was a formal partner in this research and innovation action, and its lead representative on the project, April Tash, provided critical feedback during the development and application of the conceptual framework in South Africa, Lithuania, and Serbia from approximately June 2020 until April 2021. A range of global studies on RRI undertaken during this period under the auspices of the RRING project (e.g., see Foulds *et al.*, 2023) highlighted the strong overlap between RRI as a concept and the RSSR as a live policy document with broad-based support.

The framework was constructed based on a close reading of the RSSR full policy text ( UNESCO, 2017) and the distilled 10 key priority areas, with the aim of establishing a robust conceptual foundation for identifying relevant sources of evidence to include in formal UNESCO Member State quadrennial reporting against the RSSR. The framework was developed by the author of this open letter by applying logical deduction, separating out the elements of compound sentences to add clarity, noting cross-referencing in UNESCO policy documentation where available, and matching the language and intentions of the key priority areas with details in the RSSR full policy text. Ultimately, this framework is built on logical argumentation, with full transparency to allow others to come to different conclusions or considerations about the linkages between the two documents. The review and implementation process did not result in significant changes to the framework.

This policy analysis document integrates these two distinct documents, with the aim of clarifying for Member States how the full policy text can be linked directly to each aspect of the 10 key priority areas. Development of this conceptual framework was prompted by feedback from the aforementioned national case studies that the 10 key priority areas on their own were not sufficiently elaborated to clarify which kinds of evidence where relevant for which key priority area.

## Using this conceptual framework

While this open letter may be relevant to anyone interested in evaluating RRI on a global scale or the RSSR policy instrument, its specific audience is UNESCO Member States and the working groups they convene to deliver expert input into the long-term RSSR monitoring and reporting processes. In particular, UNESCO Member State government representatives and working groups are encouraged to consider the categories indicated in the framework ( Jensen, 2021) to self-assess the comprehensiveness of the available evidence that has already been documented. This document is intended to be used as a worksheet by the informally constituted working groups that some countries convene so that they can prepare reporting on evaluation measures and indicators relating to the RSSR that are as comprehensive as possible.

It is understood that practical constraints will mean that most UNESCO Member States cannot address all aspects of the 10 key priority areas in full, comprehensive detail. For this reason, this conceptual framework is designed to be used selectively to target aspects of the RSSR where clarification and orientation would be helpful. That is, the framework is designed to allow national working groups to pick and choose which key priority areas they would like to consider further at any given point. This is a supporting document to aid the monitoring process and does not replace direct engagement with the key priority areas and the full text of the RSSR.

## Conclusion

Recent years have seen a surge in public support for science ( Jensen *et al.,* 2021). Research systems need to improve to be worthy of this public trust. To do this, we need to take an evidence-based approach to advancing the principles, such as inclusive and transparent research processes, that RRI and the RSSR share ( Jensen & Gerber, 2020). Establishing robust methods for evaluating progress in the development of more socially responsible research and innovation systems is essential to ensure progress. The RSSR is a useful means of advancing this effort, offering a ready-made agreement of many countries with monitoring already included. The monitoring process will continue in perpetuity, thus making it an excellent focus for aligning global efforts to create improved science systems. The conceptual framework included in the supplementary materials for this letter offers a tangible structure for advancing this global alignment into the future.

## Data Availability

Zenodo: Full Text Conceptual Framework for Monitoring Socially Responsible Research and Innovation (RRI) aligned to the UNESCO - led Recommendation on Science & Scientific Researchers. [Data set]. DOI: 10.5281/zenodo.5715729. This project contains the following underlying data: Full Text Conceptual Framework for Monitoring Socially Responsible Research and Innovation (RRI) aligned to the UNESCO - led Recommendation on Science & Scientific Researchers (detailed breakdown of each of the 10 key priority areas for monitoring the 2017 Recommendation, including quotations mapped over from the full text of the RSSR). Data are available under the terms of the Creative Commons Zero "No rights reserved" data waiver (CC0 1.0 Public domain dedication).

## References

[ref-16] Foulds C Khalid R Jensen EA : Social responsibility in research and innovation practice and policy across global regions, institutional types, and fields: Interview data and qualitative content analysis outputs revealing the perspectives and experiences of professionals [version 1; peer review: 2 approved, 1 approved with reservations]. *Open Res Eur.* 2023;3:65. 10.12688/openreseurope.15688.1 PMC10445911 37645483

[ref-15] Gerber A Forsberg EM Shelley-Egan C : Joint declaration on mainstreaming RRI across Horizon Europe. *J Responsible Innov.* 2020;7(3):708–711. 10.1080/23299460.2020.1764837

[ref-1] Jensen EA : The problems with science communication evaluation. *J Sci Commun.* 2014;13(1):C04. 10.22323/2.13010304

[ref-2] Jensen EA : The UNESCO Recommendation on Science and Scientific Researchers will transform working conditions, rights and responsibilities of researchers globally.LSE Impact Blog.2020; Last accessed 20 November 2021. Reference Source

[ref-3] Jensen EA : Full Text Conceptual Framework for Monitoring Socially Responsible Research and Innovation (RRI) aligned to the UNESCO - led Recommendation on Science & Scientific Researchers.2021. 10.5281/zenodo.5715729 PMC10445803 37645346

[ref-6] Jensen EA Gerber A : For science communication to be effective it should be evidence based.LSE Impact Blog.2020; Last accessed 20 November 2021. Reference Source

[ref-4] Jensen EA Kennedy EB Greenwood E : Pandemic: public feeling more positive about science. *Nature.* 2021;591(7848):34. 10.1038/d41586-021-00542-w 33654302

[ref-5] Jensen EA Laurie C : Doing real research: A practical guide to social research. SAGE: London.2016. Reference Source

[ref-7] United Nations Educational Scientific and Cultural Organization (UNESCO): Recommendation on Science and Scientific Researchers.2017. Reference Source

[ref-8] United Nations Educational Scientific and Cultural Organization (UNESCO): National Reporting on the 2017 UNESCO Recommendation on Science and Scientific Researchers.2021; Published 31/01/2021. Reference Source

[ref-9] United Nations Educational Scientific and Cultural Organization (UNESCO): 209 EX/18.IV: Implementation of standard-setting instruments, Part IV: Implementation of the 2017 Recommendation on Science and Scientific Researchers - Preparation for the next consultation from the Executive Board two hundredth and ninth session. 209 EX- Main series.2020. Reference Source

[ref-10] United Nations Educational Scientific and Cultural Organization (UNESCO), Legal Instruments: 1st aspect of the terms of reference of CR: examination of reports received from Member States in the framework of the implementation of UNESCO’s standard-setting instruments. (n.d); Accessed: 8 December 2021.

