# Supplementary material for: Linking policy and practice in monitoring socially responsible research and innovation (RRI): A conceptual framework to evaluate progress through the UNESCO-led Recommendation on Science and Scientific Researchers
Source: Open Res Eur. 2023 Jul 24;2:21. Originally published 2022 Feb 14. [Version 2] doi: 10.12688/openreseurope.14263.2 (PMC10445803; doi:10.12688/openreseurope.14263.2)
Supplement: Peer review history

# Peer review: Reviewer response for version 2

**DOI:** 10.21956/openreseurope.17663.r34093

**Published:** 2023-08-04

**Authors:** Simon Fielke

**Affiliations:** 1 Sustainability Pathways, CSIRO Environment, Dutton Park, Queensland, Australia

This letter is labelled a conceptual framework but there is no conceptual framework. The supplementary document is more of an expansion of the ten key themes rather than what I would consider an operational conceptual framework. 

 There are many references to the authors work (over half the reference list) and I am sure there are other relevant works concerning the science of responsible innovation as a process of practices - eg the original conceptualisations (Owen, Stilgoe, *et al*).

- *Is the rationale for the Open Letter provided in sufficient detail? (Please consider whether existing challenges in the field are outlined clearly and whether the purpose of the letter is explained)* – No
  The Open Letter suggests a conceptual framework that is not presented in the text nor in an appropriate format for me to consider it a conceptual framework. I appreciate that the format has only allowed limited representation of the conceptual framework in text, but that suggests to me this is the wrong format if the contribution of the letter cannot be made within the letter.
- *Does the article adequately reference differing views and opinions?* – No
  It is mainly self-citations when there are many relevant and seminal responsible innovation contributions overlooked – i.e. where did such thinking and practice come from and why is it important?
- *Are all factual statements correct, and are statements and arguments made adequately supported by citations?* – Partly
  See above comments – where is the conceptual framework built from and how are the ten themes operationalizable?
- *Is the Open Letter written in accessible language? (Please consider whether all subject-specific terms, concepts and abbreviations are explained)* - Yes
- *Where applicable, are recommendations and next steps explained clearly for others to follow? (Please consider whether others in the research community would be able to implement guidelines or recommendations and/or constructively engage in the debate)* – Partly
  As a researcher in this space I cannot see how I would operationalise the framework in the supplementary material.

Where applicable, are recommendations and next steps explained clearly for others to follow? (Please consider whether others in the research community would be able to implement guidelines or recommendations and/or constructively engage in the debate)

Partly

Does the article adequately reference differing views and opinions?

No

Are all factual statements correct, and are statements and arguments made adequately supported by citations?

Partly

Is the rationale for the Open Letter provided in sufficient detail? (Please consider whether existing challenges in the field are outlined clearly and whether the purpose of the letter is explained)

No

Is the Open Letter written in accessible language? (Please consider whether all subject-specific terms, concepts and abbreviations are explained)

Yes

Reviewer Expertise:

Agricultural innovation; innovation studies; human geography

I confirm that I have read this submission and believe that I have an appropriate level of expertise to state that I do not consider it to be of an acceptable scientific standard, for reasons outlined above.

# Peer review: Reviewer response for version 2

**DOI:** 10.21956/openreseurope.17663.r34089

**Published:** 2023-07-31

**Authors:** Steven Umbrello

**Affiliations:** 1 Department of Philosophy and Education Sciences, University of Turin, Turin, Italy; 2 Center for Religious Studies, Fondazione Bruno Kessler, Trento, Trentino-Alto Adige/South Tyrol, Italy

**Abstract**

 The abstract is well-written, summarizing the aim, methodology, and key points of the paper, including the creation of a conceptual framework for monitoring socially responsible research and innovation (RRI) in alignment with UNESCO’s Recommendation on Science and Scientific Researchers (RSSR). The importance of fostering global learning and improvement in RRI is clearly articulated.

**Introduction**

 The introduction offers an adequate overview of the international accord RSSR and its alignment with the RRI concept, emphasizing the importance of monitoring and evaluation. A clear connection to EU policy and research regarding RRI is presented, but it may still need a bit more clarity in framing the relationship between RRI and RSSR as per reviewer two's comments.

**Context for conceptual framework**

 This section details the framework's design, tone, and intended audience. While it is informative and extensive, it might still benefit from a brief summary or visual representation (like a diagram) of the framework itself in the main body to address reviewer one's concern about structure.

**Development of the conceptual framework**

 The development process of the framework is explained, including the involvement of UNESCO and the logical deductions applied in its creation. While comprehensive, the absence of the detailed framework in the main text might still leave some readers wanting a more concrete understanding of what it contains.

**Using this conceptual framework**

 This section provides practical guidance on how to use the framework, targeting specifically UNESCO Member States. It's clear, informative, and engages the reader with a specific call to action.

**Conclusion**

 The conclusion highlights the need for evidence-based approaches to enhancing RRI principles, emphasizing the importance of the RSSR and the ongoing monitoring process. The author has addressed the initial concern about redundancies.

**Author's Response**

 The author's response indicates they have taken the initial reviews seriously, making adjustments where feasible and explaining why other changes were not made. The clarification regarding the removal of the framework's content due to editorial requirements is important but leaves an unresolved concern about the structure.

**Recommendations for Improvement**

1. **Clarify the Relationship Between RRI and RSSR**: As suggested by reviewer two, the introduction should provide a clearer framing of how RRI aligns with RSSR.
2. **Include a Brief Overview or Visual of the Framework**: While it's clear the details of the framework were moved to supplemental material due to editorial guidelines, a brief summary or visual representation could enhance reader comprehension without overwhelming the main text.

**Conclusion**

 The revised manuscript has made efforts to address the concerns raised by the initial reviewers. While some improvements have been made, further refinement could enhance the clarity and comprehensibility of the paper, especially in the context of the relationship between RRI and RSSR, and the visualization or summarization of the framework. The paper represents an essential contribution to the understanding of RRI and offers a valuable tool for UNESCO Member States and others in their monitoring efforts.

Where applicable, are recommendations and next steps explained clearly for others to follow? (Please consider whether others in the research community would be able to implement guidelines or recommendations and/or constructively engage in the debate)

Yes

Does the article adequately reference differing views and opinions?

Yes

Are all factual statements correct, and are statements and arguments made adequately supported by citations?

Yes

Is the rationale for the Open Letter provided in sufficient detail? (Please consider whether existing challenges in the field are outlined clearly and whether the purpose of the letter is explained)

Yes

Is the Open Letter written in accessible language? (Please consider whether all subject-specific terms, concepts and abbreviations are explained)

Yes

Reviewer Expertise:

RRI, value sensitive design, and ethic-by-deisgn approaches to novel and transformative technologies

I confirm that I have read this submission and believe that I have an appropriate level of expertise to confirm that it is of an acceptable scientific standard.

# Peer review: Reviewer response for version 1

**DOI:** 10.21956/openreseurope.15387.r30263

**Published:** 2022-10-21

**Authors:** Raul Tabares

**Affiliations:** 1 Basque Research and Technology Alliance (BRTA), Derio, Spain

Dear author,

 Thank you for submitting your work to Open Research Europe.

 The topic that addresses this letter is relevant and timely and it tries to engage with ongoing efforts and strategies for strengthening science-society interactions. The open letter pays attention to the synergies between RRI and the UNESCO “Recommendation on science and scientific researchers’ (RSSR) that was ratified by 195 UNESCO Member States in 2017. The article is well-written, and the language employed is accessible.

 However, there are several issues that demand attention from the author to improve this open letter. Some of them are related with the content whilst others are more related with the format. In this sense, I tend to agree with the other prior reviews in several issues. I propose to the author several changes in order to strength this manuscript:

- It is important to frame the “RRI discourse” on the text and to illustrate which synergies with RSSR have. It is hard to understand for a not-specialized reader.
- Eliminate redundancies and repetitions. Especially in the conclusion which does not point to next steps or future challenges ahead.
- The introduction is too long for a short letter like this. It needs to be shortened around half (or even more) and incorporate the RRI concept which is not explained at all in the paper.
- Section two of the letter should provide more details about how the framework was set up. Specially of interest for the reader should be to expand the 10 points of the figure into subsections (more space for the figure will be welcome in the text).
- Section three will benefit from a further clarification about the “supporting function” that this document has.
- Mentions to COVID-19 at the end of the text are confusing for the reader. It should be erased.
- The text will benefit from “citation diversity”. Especially for situating the concept of RRI into the text and helping to the reader to understand the synergies between RRI and RSSR.

The changes proposed here demand significant work on this paper (major changes), but I think that the letter will benefit from this work to become a valuable contribution on this matter.

Where applicable, are recommendations and next steps explained clearly for others to follow? (Please consider whether others in the research community would be able to implement guidelines or recommendations and/or constructively engage in the debate)

Partly

Does the article adequately reference differing views and opinions?

No

Are all factual statements correct, and are statements and arguments made adequately supported by citations?

Partly

Is the rationale for the Open Letter provided in sufficient detail? (Please consider whether existing challenges in the field are outlined clearly and whether the purpose of the letter is explained)

Partly

Is the Open Letter written in accessible language? (Please consider whether all subject-specific terms, concepts and abbreviations are explained)

Yes

Reviewer Expertise:

RRI, RI, science-society interactions, STS

I confirm that I have read this submission and believe that I have an appropriate level of expertise to confirm that it is of an acceptable scientific standard, however I have significant reservations, as outlined above.

# Peer review: Reviewer response for version 1

**DOI:** 10.21956/openreseurope.15387.r28668

**Published:** 2022-04-11

**Authors:** Kutoma Wakunuma

**Affiliations:** 1 Centre for Computing and Social Responsibility, De Montfort University, Leicester, UK

This is a well articulated article written in language that is accessible. The article addresses the need for the inclusion of socially responsible research and innovation in UNESCO’s RSSR quadrennial monitoring process. It gives a clear rationale for the need for a conceptual framework intended to monitor the development of socially responsible research and innovation systems. Although the article’s language is accessible with subject-specific terms such as RSSR explained and abbreviations given, the concept of RRI could have benefited from an in-depth explanation of what it is and what it entails particularly for stakeholders unfamiliar with the term or with its practices. Further, in as far as RRI is concerned, the paper could have done well to offer differing views and opinions of RRI which may not necessarily be a universal concept that is understood and practiced in the same way across the 195 UNESCO member states. As such, different views and opinions, particularly in as far as its understanding and practice in different geographical locations are concerned would have been valuable. Therefore, given that there are 195 member states who may or may not fully understand the rationale or concept of RRI or who no doubt may practice it differently, a presentation of competing assertions would have been an ideal consideration in the article. By so doing, the article would have given insight to potential challenges in the field, therefore providing possible considerations on how to address evaluation challenges and potential mutual learning points in the monitoring process of RSSR. Further, such considerations could offer a much deeper understanding of the challenges that have prompted the need for the incorporation of socially responsible research and innovation systems when it comes to RSSR. This is important, particularly for stakeholders unfamiliar with RRI.

 The article makes a great point about UNESCO’s omission, albeit deliberately, in providing specifics of how to assess and improve the social responsibility of science systems in line with the RSSR policy instrument. Although the intention was to allow context-appropriate adaption of RRI, it would still have been beneficial to have some criteria or as indicated by the author specifics on the assessment and RRI systems. This could avoid unwieldy interpretations, implementations, and evaluations of the RSSR to the extent that the varied interpretations become challenging in their address of RSSR policy, especially in terms of comparability between member states. Similarly, the fact that member states can use the conceptual framework selectively to target aspects of the RSSR where clarification and orientation would be helpful may not bode well for comparability between member states. Granted, this is intended to be helpful for those member states who may not be able to address all 10 key priority areas in full. Despite this, it would have been beneficial to have compulsory aspects of the RSSR that each member state would need to address as a way of ensuring uniformity while leaving some as optional in the event of a failure in addressing all 10. That said, this is a timely article that is useful in its contribution to the importance of having socially RRI systems in RSSR policy.

Where applicable, are recommendations and next steps explained clearly for others to follow? (Please consider whether others in the research community would be able to implement guidelines or recommendations and/or constructively engage in the debate)

Partly

Does the article adequately reference differing views and opinions?

No

Are all factual statements correct, and are statements and arguments made adequately supported by citations?

Yes

Is the rationale for the Open Letter provided in sufficient detail? (Please consider whether existing challenges in the field are outlined clearly and whether the purpose of the letter is explained)

Partly

Is the Open Letter written in accessible language? (Please consider whether all subject-specific terms, concepts and abbreviations are explained)

Yes

Reviewer Expertise:

Information and communication technologies for development; Emerging Technologies; Computer Ethics; Gender; Responsible Research and Innovation

I confirm that I have read this submission and believe that I have an appropriate level of expertise to confirm that it is of an acceptable scientific standard.

# Peer review: Reviewer response for version 1

**DOI:** 10.21956/openreseurope.15387.r28688

**Published:** 2022-03-18

**Authors:** Justus Wesseler

**Affiliations:** 1 Agricultural Economics and Rural Development, Wageningen University, Wageningen, The Netherlands

The open letter is about suggesting a conceptual framework for monitoring RRI under the UNESCO-led Recommendation on Science and Scientific Researchers. The open letter is difficult to follow. The author suggests a framework for monitoring the RRI. The framework has been attached under supplementary information. The letter needs to be substantially improved. A few suggestions: 

- The author writes a lengthy introduction (almost half of the text), but the reader receives almost no information about what the monitoring framework is about. This needs to be added to the main body of the text. At least a summary about the key aspects of the framework one would expect in the main body of the text.
- The introduction should be shortened to about a quarter of the text.
- The open letter includes a number of redundancies. The conclusion restates what has been written in the introduction. Redundancies need to be removed.
- In the conclusion, it should be more clearly stated what the next steps according to the author should be.
- The last paragraph talks about ecosystem research and COVID-19. As a reader, I got lost. This had not been mentioned before, why now?

Where applicable, are recommendations and next steps explained clearly for others to follow? (Please consider whether others in the research community would be able to implement guidelines or recommendations and/or constructively engage in the debate)

Partly

Does the article adequately reference differing views and opinions?

Partly

Are all factual statements correct, and are statements and arguments made adequately supported by citations?

Partly

Is the rationale for the Open Letter provided in sufficient detail? (Please consider whether existing challenges in the field are outlined clearly and whether the purpose of the letter is explained)

Partly

Is the Open Letter written in accessible language? (Please consider whether all subject-specific terms, concepts and abbreviations are explained)

Partly

Reviewer Expertise:

Agricultural, environmental, and natural resource economics. Substantial experience in reviewing interdisciplinary research proposals. Expertise in developing and assessing monitoring frameworks.

I confirm that I have read this submission and believe that I have an appropriate level of expertise to state that I do not consider it to be of an acceptable scientific standard, for reasons outlined above.
